# Frailty and Colorectal Surgery: Review and Concept of Cancer Frailty

**DOI:** 10.3390/jcm12155041

**Published:** 2023-07-31

**Authors:** Hiromichi Maeda, Michiko Takahashi, Satoru Seo, Kazuhiro Hanazaki

**Affiliations:** Department of Surgery, Kochi Medical School Hospital, Kohasu, Oko-cho, Nankoku 783-8505, Japan; michikot@kochi-u.ac.jp (M.T.); rutosa@kochi-u.ac.jp (S.S.); hanazaki@kochi-u.ac.jp (K.H.)

**Keywords:** gastrointestinal surgery, cancer frailty, prehabilitation, decision making

## Abstract

Frailty is characterized by reduced physiological reserves across multiple systems. In patients with frailty, oncological surgery has been associated with a high rate of postoperative complications and worse overall survival. Further, given that cancer and frailty can co-exist in the same patient, cancer and cancer-related symptoms can rapidly accelerate the progression of baseline frailty, which we have termed “cancer frailty”. This distinction is clinically meaningful because the prioritization of interventions and the treatment outcomes may differ based on health conditions. Specifically, in patients with cancer frailty, improvements in frailty may be achieved via surgical removal of tumors, while prehabilitation may be less effective, which may in turn result in delayed treatment and cancer progression. In this review, we focused on challenges in the surgical treatment of non-metastatic colorectal cancers in patients with frailty, including those related to decision making, prehabilitation, and surgery. Potential recommendations for treating patients with cancer frailty are also discussed.

## 1. Introduction

The colon and rectum constitute the last part of the intestinal tract, the primary functions of which include the absorption of water and electrolytes and the temporal storage of feces before defecation [1,2]. Local progression of colorectal cancer manifests as hematochezia, changes in bowel habits, obstruction, and even perforation. Non-specific symptoms, such as fatigue, loss of appetite, and weight loss, are also common features of colorectal cancer. Lymphatic or hematogenous spread can lead to organ dysfunction at metastatic sites, which can ultimately result in mortality. Surgical resection has been adopted as a curative approach when appropriate [3]. However, post-resection functional decline may result in loose stools, frequent evacuation, incontinence, and stoma development, the severity and frequency of which vary depending on the tumor site.

Despite upward trends in the incidence of colorectal cancer in younger patients [4], the disease is more common in older adults. Approximately 40% of patients with colorectal cancers in developed countries are at least 75 years of age [5,6], and the age-specific incidence of colorectal cancer is known to increase with age [7]. Given both the chronological and physiological aging of the global population [8,9], emphasis on establishing a management strategy for older patients with frailty who have been diagnosed with colorectal cancers has increased [10]. Studies examining the accuracy of measurement tools for frailty have clarified the adverse effects of frailty on outcomes after colorectal surgery. Recent research has also highlighted the value of preoperative physical enhancement (i.e., “pre-habilitation”) for improving frailty status [11,12]. The available evidence emphasizes the need for clinicians to implement geriatric assessments in daily practice, the pivotal role of prehabilitation, and the importance of enhanced recovery after surgery (ERAS) pathways [13].

Nevertheless, surgical treatment of non-metastatic colorectal cancer in patients with frailty is associated with many challenges, even after implementing appropriate measurement tools. In contrast to previous reviews, our exploratory review aimed to focus on practical issues concerning decision making in patients with frailty, the prognosis of colorectal cancer without surgical treatment, and practical issues concerning prehabilitation from a surgeon’s perspective; introduce the concept of “cancer frailty”—a pretreatment health status characterized by a rapid progression of baseline frailty caused by cancer and cancer-related symptoms—to aid the development of treatment plans for this population; and introduce tentative treatment approaches based on frailty status.

As evidence for frailty among patients with colorectal cancer remains sparse and fragmented at present, we also refer to evidence from other fields of surgery/oncology, which can be considered a limitation of this study.

## 2. Physical Frailty

Physical frailty is a dynamic medical state characterized by decreased strength, endurance, and physiological function, as well as increased vulnerability [14]. External stressors lead to greater damage in patients with frailty, who tend to experience delayed or incomplete recovery of health status. Although frailty is common in the older adult population [15,16], it is not a direct result of aging. As frailty affects multiple physiological systems to various degrees, the population of patients with frailty is heterogenous, necessitating a comprehensive assessment and personalized treatment approach in clinical practice. Notably, several studies have demonstrated that the progression of frailty can be slowed or reversed [17,18], indicating that frailty is an actionable preoperative variable.

Objective assessments of frailty are necessary when designing and implementing treatment strategies. In 2001, Fried et al. reported a relationship between physical function and health problems, such as incidental falls, decreased morbidity, and decline in activities of daily living (ADL), based on an analysis of data from 5317 older adults included in the US Cardiovascular Health Study (CHS) [19]. Based on their analysis, the authors operationally defined frailty as “a clinical syndrome in which three or more of the following criteria were present: unintentional weight loss (10 lbs. in past year), self-reported exhaustion, weakness (grip strength), slow walking speed, and low physical activity”. Subsequent studies have demonstrated its suitability for predicting surgical outcomes [20]. Furthermore, information obtained via frailty assessments has been utilized for clinical and surgical decision making and for preoperative risk reduction. To date, numerous assessment tools have been developed, validated, and utilized in research and clinical practice. However, the most valuable tools for patients with colorectal cancer remain to be established.

## 3. Measurement

### 3.1. First Impression

The first clinical impression, the intuition of medical staff, works well when evaluating frailty and predicting prognosis [21]. Although their study was conducted outside the field of cancer necessitating abdominal surgery, O’Neill et al. demonstrated that the judgment of frailty at the first encounter represents a valuable alternative indicator of overall survival among patients requiring vascular surgeries. The authors prospectively recorded first impressions for each patient and analyzed their medical records over four years. Among 392 patients evaluated, 133 deaths occurred during the study period, and the hazard ratio (HR) was significantly higher in the frailty group than in the non-frailty group (HR: 2.14, 95% confidence interval: 1.51–3.05). O’Neill et al. aimed to determine whether the first impression could be used to determine each patient’s suitability for the proposed operation before obtaining medical information. Thus, the study aim did not directly focus on frailty. Despite additional weaknesses in reproducibility and objectivity, their study provided unique insight into the value of adding these variables to improve prognostic accuracy.

### 3.2. Measurement of Frailty and Comprehensive Geriatric Assessment (CGA)

To date, “gut feelings” have been considered too ambiguous to be incorporated into the treatment plan. Furthermore, such a subjective measure is often insufficient to detect non-severe frailty. Clinical practice guidelines for the management of frailty recommend identifying frailty using validated tools [22]. Among these, an expedient instrument must be chosen under specific clinical circumstances. For instance, the Clinical Frailty Scale (CFS), which divides the patient’s condition into categories from 1 (robust health) to a higher number (severely frail) [23], can be used to predict adverse outcomes after surgery [24] and is advantageous because it requires minimal training [25]. However, there is a low concordance rate in identifying patients with frailty using different instruments, as shown in Venn diagrams [26,27,28]. For instance, a recent study of 151 patients undergoing hemodialysis reported that the CFS and FRAIL scales identified frailty in 19 and 50 patients, respectively, with only 14 patients having been diagnosed with frailty using both instruments [27].

CGA includes a measurement of frailty and has been considered the reference standard [29]. This multidimensional, interdisciplinary assessment is aimed to clarify the type and extent of support required [30], and frailty measurement followed by CGA is often utilized in clinical settings. However, this strategy has yet to be proven efficient; frailty screening followed by comprehensive assessment does not avoid unnecessary assessment [31]. However, the barrier in applying the “GA-for-all strategy” [31] is an understandably high burden due to limited human resources. A panel of multidisciplinary experts recommends the implementation of simple tools into daily clinical practice without delay [32].

## 4. Decision Making for Patients with Frailty

### 4.1. Complexity and Uncertainty

Among the difficulties in selecting a surgical strategy for patients with frailty is that the benefit of surgery is limited without a sufficient relative increase in longevity. The more “frail” the patient, the more severe and frequent the surgical complications. In addition, functional decline after surgery (use of stoma, incontinence, and frequent stools) and financial burden influences the decision to opt out of surgery. In contrast, the symptoms of cancer may lead one to undergo surgery, considering that it is more rational to undergo treatment earlier rather than later, at which point greater functional decline will have occurred. In such cases, surgery can be encouraged when there is a reasonably high possibility of cure. The recent development of a management strategy to optimize health status before surgery (prehabilitation) adds complexity to the overall picture of the decision-making process. Herein, we refer to two articles relevant to the practice.

### 4.2. Older Patients with Frailty

Puts et al. investigated how frailty affects the decision to receive or rejectchemotherapy [33]. The authors collected pathological data for 29 patients with breast, colorectal, and lung cancers who were indicated for palliative chemotherapy. Using semi-structured interviews and surveys, they found that older patients overwhelmingly respected the opinions of their oncologists, as represented in ratings related to “trust in my oncologist.” In addition, older adult patients underwent chemotherapy, given the desire to prolong their lives. These results were consistent with those of a systematic review highlighting the physician’s recommendation as a key factor in deciding cancer treatment in older adults [34]. The authors also found that comorbidity, frailty, and unwanted effects of treatment exerted only a marginal influence on their decisions. Both younger and older patients are eager to avoid functional and cognitive decline and maintain their quality of life [35,36,37], especially when critically ill. However, this study clarified that the desire to remain alive is fundamentally important in the decision to pursue treatment.

### 4.3. Heterogeneity of the Surgeon’s Preference

A study investigating treatment preferences among surgeons in hypothetical clinical scenarios has yielded alarming results. Daniels et al. prepared 18 experimental cases with differences in diagnosis, age, cognitive impairment, functional status, and comorbidities [38]. They found that the decision to offer surgery significantly differed among surgeons; several scenarios had a decision concordance rate of less than 85%. In the context of the study by Puts et al. [33], blindly following a surgeon’s discretion is not recommended. In the Daniels study, severe comorbidities, age > 85 years, and severe cognitive impairment (all considered related to frailty) were statistically significant predictive factors for non-operative treatment. We agree with their proposition that the diversity of surgeons’ preferences must be reduced by alerting policymakers [38]. Furthermore, treatment preferences can translate to actual differences in the provision of surgical care, as observed, given disparities in the resection rates of non-metastatic colon cancer among hospitals [39]. In addition, these results highlight the need “to avoid framing the information towards the surgeon’s preference” [40], which is the initial step of shared decision making, as well as the importance of a multidisciplinary cancer board.

### 4.4. Changes in Decisions after Geriatric Assessment/Consultation

Geriatric assessment and consultation have been shown to impact treatment decision making [41]. Less invasive treatments are primarily selected when such changes are made, although some patients also decide to accept more invasive treatment after geriatric assessment/consultation [42,43]. Consequently, adverse events due to appropriate cancer treatments are likely to be reduced.

## 5. Choosing Non-Surgical Treatment

Although related research is scarce, information regarding non-operative clinical outcomes is also necessary for patients to determine their own treatment [44]. However, patients who reject surgical treatment have severe comorbidities and frailty, which can negatively affect overall survival. Even with a mild decline in health status, non-metastatic colorectal cancer extending to adjacent organs and requiring combined organ resection can remain untreated. In such cases, the survival rate with non-surgical treatment tends to be poor. Thus, the interpretations of the referenced studies [44,45,46,47,48,49,50] require caution and should not be misused to guide patients and families to undergo surgery (Table 1).

A large study of 20,423 patients aged >70 years with colorectal cancer was conducted in the Netherlands [45]. In that study, nearly 5% of patients with colon cancer opted not to undergo surgery. Over a median period of 33 months, the mortality rate in patients with colon cancer who underwent surgery was 28%, versus 91% in those who did not. Further, the 3-year relative survival rate was only 9% for non-surgical patients, while it was 91% in post-surgical patients [45]. The authors argued that the spread of shared decision making in recent years may be responsible for the increasing frequency of non-surgical treatment, which is an alarming proposition. Franklyn et al. analyzed data for 407 patients aged ≥80 years who were treated for stage I–III colorectal cancer between 2007 and 2015. In total, 132 patients did not undergo surgical resection. The 2- and 5-year overall survival rates in the non-surgical group were 38.9% and 11.3%, respectively, which were significantly lower than those in the surgical group [46]. The authors also noted that only one-third of these patients underwent a formal anesthetic assessment concerning fitness/unfitness for surgery. Based on an analysis of data from 31,574 patients over 80 years of age diagnosed with stage I–III colon cancer between 1992–2005, Neuman et al. noted that many older adults with colon cancer in the US die from unrelated causes [50]. In their study, the 1-year overall survival rate for patients who did not undergo tumor resection was 56%, whereas the disease-specific survival rate was 76% (*n* = 5665).

## 6. Colorectal Cancer Surgery in Patients with Frailty

### 6.1. Short-Term Results

Frailty is associated with higher rates of postoperative complications, short-term mortality, readmission, and increased length of hospital stay [51]. Furthermore, frailty is an independent risk factor for anastomotic leakage after colectomy, and patients with frailty are more prone to death from anastomotic leakage or other complications than those without [52]. However, quality of life may not be affected [53].

### 6.2. Overall Survival and Disease-Specific Outcomes

The impact of surgery may be greater than previously considered given that 1-year excessive mortality is far higher than 30-day mortality, especially in patients with comorbidities, higher disease stage, emergency surgery, and postoperative surgical complications [54]. As frailty is associated with delayed recovery from injury, long-term outcomes tend to worsen after surgery. In addition, several studies have demonstrated poor overall survival following surgery in patients with frailty [55,56], which has been related to a “natural” decline in function.

The effect of frailty on disease-specific survival remains controversial. Neuman et al. investigated the factors associated with overall and disease-specific survival in patients with colorectal cancer over 80 years of age using the SEER-Medicare Database. Briefly, they identified 31,574 patients who had been diagnosed with colon cancer between 1992 and 2005. One-year colon cancer-specific survival was 89% for patients with curative resection and 76% for patients with non-surgical treatment. Patients with frailty accounted for 6.8% of the surgical group and 14.6% of the non-surgical group. Multivariate analysis revealed that being frail is an independent predictive factor of cancer-specific death (adjusted hazard ratio: 5.24, 95% confidence interval: 4.6–5.97, *p* < 0.005). However, a significant limitation of the study was the lack of information on cancer stage, although the authors claimed that the impact of adding cancer stage had minimal effects on the model [50].

More recently, Mima et al. evaluated 729 consecutive patients to compare stage I–III colorectal cancer outcomes between those with and without frailty [24]. After adjusting for clinical variables, frailty was associated with recurrence-free survival and overall survival. The authors cited chronic inflammation and antitumor immune responses as plausible mechanisms underlying poor resistance to tumor recurrence in the frailty group. Indeed, the relationships among frailty, immunological markers, and survival, as well as those among frailty, inflammation, and low survival, have been statistically analyzed in patients with colorectal and other cancers [57,58]. However, further studies are required to clarify whether frailty affects cancer progression through these molecular mechanisms. The rate of recurrence varies greatly even among patients within the same stage [59,60], and the agent used for chemotherapy, especially the presence/absence of oxaliplatin, may impact outcomes. In addition, patients with frailty may experience reduced tolerability to chemotherapy, as discussed by authors [24]. A study have also reported no difference in disease-specific survival between patients with and without frailty following colorectal surgery [61]. Given that the addition of frailty status does not improve the accuracy of models for predicting disease-free or disease-specific survival [62], a recent study also denies that there are direct effects of frailty on tumor recurrence. Therefore, the impact of frailty on disease-free survival requires further investigation.

## 7. Prehabilitation

Preoperative multimodality optimization for older patients with colorectal cancer includes exercise training, nutritional therapy, psychological intervention, and smoking/alcohol cessation [10]. Observational and retrospective studies have suggested the benefit of prehabilitation (especially physical exercise) over usual care in reducing postoperative complications after colorectal cancer surgery [63,64]. This is in accordance with evidence from systematic reviews in the field of cancer surgery [65,66]. In addition to its effects on postoperative complications, prehabilitation can help to prevent functional decline, which may influence the treatment selection for older patients.

Once frailty has been identified and surgical treatment has been scheduled, surgeons generally become more interested in reversing frailty to obtain better outcomes. However, significant obstacles have emerged for both patients and surgeons. The first constraint is time. In Japan and the US, the median interval between diagnosis and initial treatment is approximately 4 weeks [67,68]. Essential evaluations of frailty, the decision-making process leading up to treatment, agreement to undergo prehabilitation, rehabilitation consultations, and re-evaluation of fitness for the scheduled treatment ultimately result in a substantial waiting time prior to operation. Operations may also need to be rescheduled because “frailty [can] not always be improved” [69]. Generally, a 4-week delay in treatment is associated with a slight yet significant decline in overall survival in patients with colorectal cancer [70]. The fear of delays in treatment by both surgeons and patients is understandable. In this regard, Franssen et al. advocated for a shift toward optimization to prevent adverse surgical outcomes in those with frailty and other high-risk patients [71].

Recently, Cuijpers et al. have discussed the low feasibility of prehabilitation in the context of colorectal cancer [11]. Their systematic review evaluated the generalizability and feasibility of exercise in this population, although the authors noted that only a few potentially eligible patients were included in the trials examined. Furthermore, the retention rate of the patients in these studies ranged from 18.4% to 58.2% [11]. A clinical study from Australia was included in the systematic review, which demonstrated representative results for low recruitment [72]. The authors found four main factors contributing to non-involvement: psychological reasons related to large bowel function and frailty, psychological reasons related to family burden and frailty, timing of recruitment (after receiving bad news), and logistic reasons. Socioeconomic status has also been associated with participation in prehabilitation [73]. These issues represent problems to be addressed not only in clinical trials, but also in daily clinical practice. Potential solutions to such problems include personalized prescription of exercise adjusted for individual health status, ensuring the patient’s understanding of the potential benefit of prehabilitation based on a timely explanation, addressing psychological burden in both patients and family members, and community-based interventions [74,75].

## 8. Cancer Frailty

**Figure 1:**
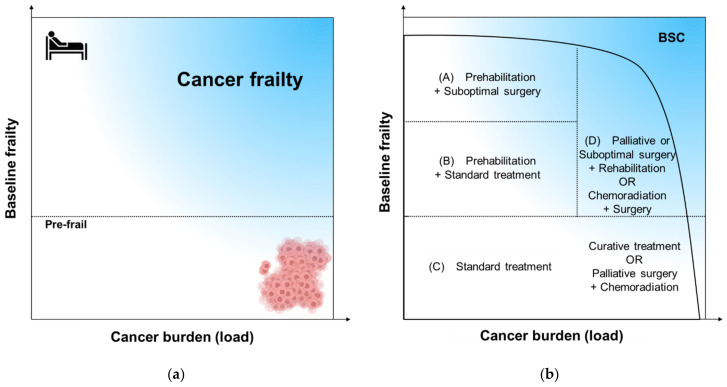
Graphical overview of the concept “cancer frailty” and possible treatment options. (**a**) Cancer frailty. Cancer frailty is a concept used to emphasize that the cancer burden (load) accelerates the progression of baseline frailty, necessitating more complex treatment. Cancer frailty is located on the plane that unfolds along two axes and is mainly located in the right upper area. (**b**) Proposed treatment for non-metastatic colorectal cancer. Standard treatment options should be considered for fit patients without excessive tumor burden (load) (Area C). Meanwhile, patients with moderate frailty may benefit more from a combination of prehabilitation and standard cancer treatment. Finally, in patients with severe frailty, improvements in health status with prehabilitation may not allow for curative resection. (Area A&B). Furthermore, in patients whose health status has markedly deteriorated due to excessive tumor burden, implementing standard treatment may be difficult. In such cases, symptomatic treatment (decompression of the colon, anemia treatment, chemoradiation) may precede curative treatment (right border of Area C). For patients with cancer frailty, the benefits of nutritional support and exercise may be limited unless the underlying cause is completely resolved. Thus, palliative resection followed by rehabilitation is appropriate for some patients within this group.

We propose the term “cancer frailty” to describe the preoperative frailty level. This term is useful for characterizing the health status of patients with solid cancer and baseline frailty and aiding the development of treatment strategies. Cancer frailty does not simply refer to the coexistence of cancer and frailty in one patient; rather, the term is used to emphasize the important clinical observation, such as when cancer burden (load) accelerates the progression of baseline frailty, necessitating more complex treatment. Cancer frailty does not fall solely within the spectrums of non-frailty and frailty or non-cancer and excessive cancer burden; rather, it exists on the plane that unfolds along these two axes (Figure 1a).

Frailty is associated with multiple health-related problems, including cancer, polypharmacy, cognitive disturbance, mood disorders, and social vulnerability; these factors exacerbate impairments in an individual’s health status [76]. Goede et al. claimed that the relationship between frailty and hematological malignancy is bidirectional, indicating that frailty affects the outcomes of hematological cancers. Conversely, cancer and cancer treatment have been shown to affect frailty [77]. A similar relationship exists between solid cancers and frailty. Cancer-related symptoms influence health status by enhancing the impact of each component in the vicious cycle of frailty [19]: fatigue due to anemia, weight loss due to appetite loss, and energy exhaustion due to cancer metabolism. The impact of cancer burden and cancer-related symptoms should theoretically be more profound in patients with frailty than in those without frailty because of reduced resilience to external stressors [14,76]. Thus, cancer frailty is essentially consistent with frailty and an extraction of vital components of the whole picture.

Based on extensive surgical experience, many surgeons have noted that the impact of treatment on the patient’s condition depends on the patient’s pretreatment status, the extent of the cancer burden and symptoms, and the duration and invasiveness of treatment. Postoperative adjuvant chemotherapy may worsen ADL function in patients with prefrailty or frailty after several courses of treatment. Meanwhile, removal of the cancer may result in a prompt improvement in frailty status, which is paradoxical, given that surgical invasion is a strong acute stressor.

## 9. Management and Future Directions

**Figure 2:**
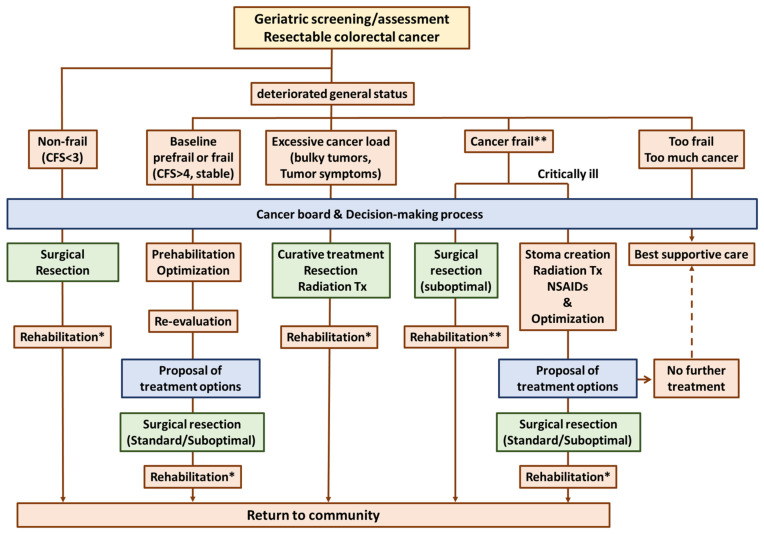
Flowchart of treatment with consideration for frailty. Typically, patients without frailty tend to choose curative cancer treatment over other treatment approaches (left side). However, it is crucial to acknowledge that even this population may develop frailty after prolonged hospitalization due to postoperative complications, highlighting the need for rehabilitation and assessment of necessary support (*). Deterioration of health status can be divided into at least four major categories: baseline frailty, excessive cancer load, cancer frailty, and too frail/too much cancer. In patients with cancer frailty, prehabilitation may not lead to improvements in frailty, and patients and surgeons may find it challenging to strike a delicate balance between curative intent and overall health. In patients with severe cancer frailty, palliation of symptoms via stoma creation, radiation therapy, and continuous rehabilitation (optimization (**)) can be initially chosen, and subsequent improvements in frailty may enable surgical resection in the future. Notably, the decision-making process extends beyond the diagnosis and should be thought of as continuous and dynamic, as changes in clinical decisions are not uncommon. Abbreviations: CFS, clinical frailty scale; Tx, treatment.

Based on the findings of our narrative review and the novel concept of cancer frailty, we have provided a summarized potential treatment plan for patients with frailty and non-metastatic colorectal cancer (Figure 1b). A clear boundary has not been established among frailty, cancer burden, and cancer frailty. Additionally, comparative studies for treatment options remain scarce. These uncertainties highlight the importance of opinions generated by multidisciplinary cancer boards and the shared decision-making process (Figure 2).

In patients without frailty who have been diagnosed with early-stage colorectal cancer, a standard treatment plan should be considered. In addition, postoperative adjuvant chemotherapy or preoperative chemoradiotherapy (followed by systemic chemotherapy) for rectal cancer should be considered in this population. Among patients with an excessive cancer burden, for whom surgical resection is still indicated, surgical treatment may be considered to avoid postoperative complications. Bulky tumors occupying the pelvic cavity can be effectively reduced with preoperative chemoradiotherapy, leading to symptom alleviation and margin-negative resection. For patients with frailty without cancer symptoms, prehabilitation is often planned. The interval between diagnosis and surgery should not be considered as the treatment delay for this group, but should be interpreted as the optimization period [71]. However, given that frailty is not always reversible, the extent of resection should be determined based on the frailty level after the second evaluation. Importantly, prehabilitation may be less effective than expected in this population because the cancer burden (load) can impede the restoration of strength. Thus, obsessing over prehabilitation may result in a missed opportunity for treatment.

Early removal of the tumor or palliation of symptoms with a stoma (if appropriate), bypass, and combined treatment with NSAIDs and palliative radiation may dramatically improve the patient’s condition and open the door to further treatment options. Occasionally, the best supportive care may be the most appropriate option for patients in this group.

Ultimately, the concept of cancer frailty could be helpful for assessing health status and designing treatment plans. However, an operational definition for isolating patients with cancer frailty remains to be established. Future studies should aim to thoroughly analyze preoperative variables and the trajectory of frailty scores shortly before diagnosis, immediately after prehabilitation, and after surgery to aid in establishing such a definition.

## 10. Conclusions

We reviewed the literature on the surgical treatment of colorectal cancer in patients with frailty, focusing on the practical difficulties. The evidence for approaches addressing these challenges remains sparse, and further studies are required to improve the treatment outcomes in this population. We proposed the term of “cancer frailty”, which reflects a health status in which cancer rapidly worsens baseline frailty. This concept can aid in interpreting assessment results and selecting a plan for overall treatment, and promote clinical decision making.

## Figures and Tables

**Table 1:** Consequences of non-surgical treatment of colorectal cancers.

Investigator	Data	Patients	Stage	n	Outcomes
van der Vlies E., et al. [45]	Cancer registry of Netherlands 2014–2018	≥70 years old	non-metastatic	Total: 27,758 Non-surgical: 2446	Colon cancer 3-year relative survival: 91% (surgical) and 9% (non-surgical) Rectal cancer 3-year relative survival: 93% (surgical) and 37% (non-surgical)
Franklyn J., et al. [46]	England tertiary care hospital 2007–2015	≥80 years old	non-metastatic	Total: 407 Non-surgical: 132	Median survival: 14.5 months (non- surgical) and 87.8 months (surgical)
Abdel-Halim M., et al. [47]	England teaching hospital 2010–2011, 2013–2015	-	I–IV	Total: 909 Too frail (for surgery): 79	2-year mortality: 19.2% (major resection), 75.9% (too frail), and 83.2% (cancer too advanced)
Bethune R., et al. [44]	England, teaching hospital 2010–2012	≥80 years old	non-metastatic	Total: 39 Non-surgical: 39	Mean survival time: 1 year and 176 days
Chen T.C., et al. [48]	National Taiwan University Hospital and its Hsinchu Branch 2001–2015	≥90 years old	I–IV	Total: 100 Non-surgical: 29	Median overall survival: 23.92 months (surgical) and 2.99 months (non- surgical)
Kim S., et al. [49]	Hallym University Sacred Heart Hospital in Korea 2007–2017	≥80 years old	I–IV	Total: 114 Non-surgical: 41	3-year overall survival: 60.7% (operative) and 9.1% (supportive care)

## Data Availability

Not applicable.
